# Ethical decision-making in biopharmaceutical research and development: applying values using the TRIP & TIPP model

**DOI:** 10.1080/21645515.2019.1700714

**Published:** 2020-01-15

**Authors:** Tatjana Poplazarova, Claar van der Zee, Thomas Breuer

**Affiliations:** Department of Medical Governance & Bioethics, GSK Vaccines, Wavre, Belgium

**Keywords:** Ethical decision making, human subject research, bioethics, values-based decision making, value-based decision making, biopharmaceutical, research and development (R&D), bioindustry ethics, decision making model, decision making tool

## Abstract

“Values-based decision-making” frameworks and models are widely described in the literature in various disciplines, including healthcare settings. However, there is a paucity of literature on the application of systematic methods or models in the biopharmaceutical research and development (R&D) field of drugs, vaccines, and immunotherapeutics.

In this report, we describe our model that uses company values along with framing questions in a five-step process to guide ethical decisions in the vaccines R&D context. The model uniquely supports practical prospective decision-making: employees are engaged as moral agents applying values and principles to guide their decision in a specific situation. We illustrate, by way of case studies, how the model is being used in practice. The consistent application of company values during decision-making calls upon employees to use their judgment, therefore reducing the need for the organization to systematically generate written instructions. Finally, we report on preliminary results of model adoption by teams within our organization, discuss its limitations and likely future contribution. We applied our model within a vaccines R&D context and believe its use can be extended to other areas where business-related decisions impact patients.

## Introduction

Values-based decision making has been defined as “*decision making based on the values of the organization and the goals these values support*”,^1^ and as organizational ethics which intentionally uses values to guide the decisions in a *“proactive and not just reactive way*”.^2,^^1^

Ethical decision-making models have been widely applied and studied in many disciplines, particularly within the clinical healthcare context.^3^ Although it is now recognized that decision making in the biopharmaceutical industry should be driven by values-based considerations,^4^ there is a paucity of literature on systematic methods or decision models applied in this industry setting that innovates and produces drugs, vaccines, and immunotherapeutics.

Although many biopharmaceutical companies publicly communicate their core organizational values,^5-8^ there may be variations in their interpretation, and their application in day-to-day decision making may not be straightforward.

In this paper, we first describe our search for evidence of models used to make ethical and values-based decisions in the context of vaccines research and development (R&D) activities in a biopharmaceutical industry setting.

We then describe a practical model developed to aid prospective decision making and illustrate its practical application, using real case studies. Next, we report on how it is being introduced to employees through workshops and summarize feedback from attendees. Finally, we discuss the potential contribution, and limitations, of such a model in the biopharmaceutical R&D setting, as well as propose ways forward for its application and development.

### Review of evidence on ethical decision making

(See search terms in Supplementary material)

There exists a substantial body of literature on ethical decision making in a wide range of disciplines (e.g., in psychological counseling, healthcare management, and in a business context), and decision-making models all tend toward a broadly similar format.^3,9-12^ However, research into decision making in the biopharmaceutical industry is limited to a survey published in 2005,^13-15^ based on in-depth interviews with personnel from 13 bioindustry companies. It includes examples of pharmaceutical companies using a variety of initiatives to encourage employees to refer to company values while making important decisions. The survey findings support our assertion that these values can play an important part in making ethical decisions in business.

A more recent framework for ethical decision making in biopharmaceutical industry R&D has been described as “a useful model for translating ethical aspirations into action – to help ensure pharmaceutical human biomedical research is conducted in a manner that aligns with consensus ethics principles, as well as a sponsor’s core values.”^16^ Although there is evidence for the usefulness of applying an ethical decision-making model to evaluate past real-life cases,^17^ a model for systematic and prospective evaluation of decision options according to values has not yet been reported.

Due to the operative nature of the biopharmaceutical R&D context, models for decision-making need to facilitate the choice of options that will result in implementable solution. We propose a practical method to prospectively guide a decision through values-assessment of options (that is, *what needs to be done* from the choice of actionable solutions). In the same way that a “moral compass tool” approach relates the understanding and interpretation of moral concepts to the situated contexts of concrete practices,^18^ our model defines and frames values in the operative context in which the decision takes place, rather than solely considering them in their abstract form on a theoretical-normative level (that is, *what ought to be done* following a certain ethical theory).

Our decision-making model is used within GSK Vaccines for resolving complex questions encountered within the R&D context that have an impact on the rights and well-being of patients and/or research participants, as well as questions around engagement with the scientific community. Such decisions are under the scope of the company’s Vaccines Medical Governance & Bioethics team and Chief Medical Officer, from product discovery until licensure and use by patients.

### Values-based model for decision making

Understanding what values mean is important for how they will be applied in decision making. Previous researchers have shown that value sets used in decision-making frameworks, and even the meanings attributed to individual values, can vary between different healthcare organizations.^19^ For example, “transparency” may be interpreted differently by different people, while if it is explicitly defined, there can be shared understanding and alignment among employees on what is meant by it and how they are expected to apply it.

In our model, GSK company values^2^ (transparency, respect, integrity and patient focus, known by the acronym TRIP) have been defined and their application facilitated by questions that help frame their meaning. For example, on what transparency means when applied to a proposed solution, by posing the framing question upfront: “How will we inform relevant stakeholders and share this decision?” the employee can reflect and assess whether their proposed action will be transparent. Other examples of framing questions for integrity, respect for people, and patient focus values are presented in Table 1.

**Table 1.** Values and framing questions.

Definition of Value^a^	Framing Questions
**Transparency**	
● Ensuring what we say or write is fair and honest, and not misleading or incomplete. ● Providing timely, relevant, and accurate information.	How will we inform relevant stakeholders? How will we share and document the solution chosen?
**Respect**	
● Actively seeking, valuing and drawing on the differing knowledge, perspectives, experience, and styles present in our global community of employees. ● Creating an atmosphere of trust, in which concerns can be fully raised.	Have all perspectives/stakeholders been considered? How does the chosen solution help to build trust externally and an atmosphere of trust within the company?
**Integrity**	
● Acting legally and fairly, within the spirit of all laws, regulations, and policies. ● Making realistic commitments and keeping our promises. ● Looking for principles, not loopholes.	Would you be comfortable to discuss the chosen solution with your family/in public? Is the solution in line with the spirit of GSK policies and compliant with applicable laws and regulations?
**Patient Focus**	
● Focusing on the patient’s and consumer’s needs in research. ● Ensuring patient/consumer safety is paramount. ● Ensuring product quality and reliability of supply.	How does this solution put the interests of the patient and/or trial participant first? How does this solution take into account any potential risk for the patient’s safety & wellbeing?

^a^https://uk.gsk.com/en-gb/careers/working-at-gsk/our-culture-and-values/.

In order to make decisions in an operative environment such as R&D, the context in which the decision takes place needs to be accounted for. We defined four contextual factors that influence the choice of decision options: Timing, Intent, Proportionality, and Perception (or TIPP, see Table 2). The implementation of a decision must take place at a *time* when there is a legitimate need for it to occur, for example deciding to donate vaccines during humanitarian crisis. The *intent* of each option needs to be clarified so that its appropriateness may be assessed, for example whether the aim of an external presentation is to communicate science or convey promotional messages. The scale of the proposed solution should be *proportional to the need*, so that the question is resolved without creating additional problems – for example, using images as a way of communicating disease awareness should not raise unjustified fear. Feasibility and cost of options are also evaluated as part of proportionality, putting in perspective the responsible use of limited resources. Finally, the solution should be checked to ensure it will be *perceived* as consistent with regard to considered timing, intent, and proportionality.

**Table 2.** Contextual factors and framing questions.

Contextual Factors	Framing Questions
**Timing**	
Legitimate need for the activity when it occurs.	Is the timing of your solution appropriate?
**Intent**	
Clear aim to do/communicate science or promotion.	Are the intentions of your solution clear and appropriate?
**Proportionality**	
Scale of solution proportionate to the need; responds to the risk without creating bigger risks.	Is the solution proportional to the situation you want to address?
**Perception**	
Solution would be perceived as consistent in terms of the timing, intent and proportionality.	Is the solution likely to be seen as consistent in terms of the timing, intent and proportionality?

### Decision making steps

Structured decision making follows a logical flow from gathering background information to generating options and evaluating them. Evaluation of options is done according to a set of objective criteria that would qualify one option as most acceptable or favorable over other options.^20-22^ *Ethical* decision making, on the other hand, sees options evaluated according to moral criteria or values.^23^ In our model we adopted company values as a basis for ethical evaluation and integrated them into the decision-making process, which aims to select the best possible option in the particular situation, taking into consideration the impacts on the various stakeholders. The integration of company values makes it easier for employees to relate to them, ensuring alignment and consistency between employees’ understanding on a personal and organizational level, as has already been described in a business context.^24^

**Figure 1.**
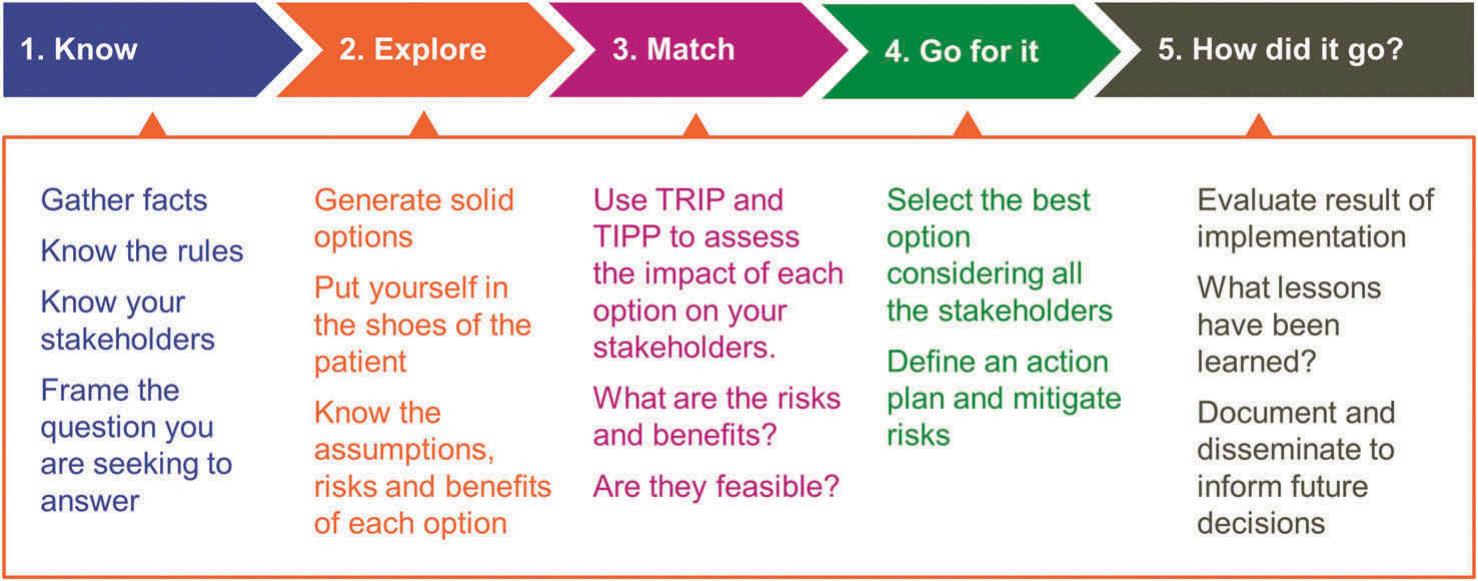
Five-step values-based decision making model.

Our decision-making process comprises five steps, and the values assessment of options is integrated in Step 3 (Figure 1). Our model has elements in common with others. It begins with the problem statement. It encourages users to put themselves in the shoes of the patient, and to check what rules and regulations apply to the specific situation.^18^ But, uniquely, our model incorporates the company values into a structured assessment of the possible solutions (Figure 1).

### Model application

Although the model may be applied to decision-making by individuals, the experience reported here is derived from application by research teams. Boxes 1 and 2 describe two real-life examples of prospective decision-making, while Box 3 illustrates a use of the model to interrogate a decision that pre-dates the implementation of the TRIP & TIPP model. When they encounter a complex question, it is usually escalated to an internal multidisciplinary bioethics board for their deliberation and decision. When using the model, members from the research team themselves generate decision options following steps 1 and 2, which they subsequently evaluate by using the values-based assessment of each option in step 3. Values assessment of options proposed by the team is concurrently performed by the board using the same model. All answers are collected by a mobile application developed for this purpose and the aggregate result of the responses is shared with the board, which includes a neutral facilitator with a relevant understanding of the business and bioethics. Deliberation is achieved following clarification on any points in which there are major discrepancies in the collected answers. Resolution is again sought in alignment with TRIP & TIPP principles.

This process allows for the so-called top-down (by bioethics board) and bottom-up (by research team) assessments to be complemented,^25^ whereby both teams and board apply the same values-based decision-making model. Employees are thus engaged as moral agents at the center of decisions, which has the potential to increase their autonomy and competence, as has been reported in healthcare settings.^26,27^

### Values-based decision making workshops: employee experience and feedback

The values-based decision-making model, known within the company as the “TRIP & TIPP model,” has so far been introduced to 956 employees in GSK Vaccines R&D with backgrounds in research, clinical, medical, safety or regulatory, through workshops in which hypothetical case studies are used to practice the methodology in a collaborative fashion. Approximately half of these attended a face-to-face session, in groups of 18 employees on average; the others took part in virtual sessions, in which groups of 24 employees on average connect online to an interactive session run by a live presenter. In both types of sessions, feedback was sought immediately afterward, and additionally by survey 6 months postsession.

In a survey of 470 workshop participants who attended face-to-face sessions held between February 2017 and May 2019, 87% responded that they would find the model helpful for decision-making. Negative – though constructive – feedback was received from 1.8%. (Response rate = 82%). Feedback following virtual sessions held between October 2018 and June 2019 gave similar results: 91% of responders found the model helpful (response rate = 79%).

At 6 months after face-to-face workshop attendance, 49% of respondents said they had used the TRIP & TIPP model after the workshop and most of them (87%) found it useful (response rate 33%). Results from a 6-month survey following virtual workshop are similar: 44% used the model and 96% found it useful, but the response rate to this survey was lower (17%).

## Discussion

This is in our opinion the first report of application and early assessment of a values-based decision-making model in the biopharmaceutical context. The novel element of the GSK Vaccines model is that, although it can be applied to both future and past cases (Boxes 1, 2, and 3), its primary purpose is to determine prospective solutions in line with company values. This distinguishes it from previously reported theoretical models that have been developed in order to test decisions already made.^17^

Like most ethical decision-making models used in different fields, our model follows the pattern of *facts gathering, option generation, option assessment according to ethical criteria, preferred option choice, and retrospective evaluation*.^20-22^ Previous research in the field of business ethics has reported the need to complement consequentialist and deontological ethics approaches with a virtue-based orientation to make the most comprehensive ethics decision model.^28-30^ The foundational elements of our model apply such a complementary approach and uniquely support practical prospective decision making: employees are engaged as moral agents applying values and principles to guide their decision in a specific situation.

Practically, our model and its application dynamics (i.e., using group deliberations) help employees make decisions in a focused, structured way, moving away from a purely intuitive approach. Moral intuition is guided by the systematic use of explicit *framing questions* to increase the understanding and clarity of values during the process of decision-making. Value application is further facilitated by an explicit definition of the context with *contextual questions*. Increasing the recognition and clarity of the values inherent to practice-based decision making has been reported to promote sound ethical reasoning.^31^ Moral intuition is further aided by the scoring of different options, which helps to detect and distinguish the ethical tension between the values that those options generate and where reasoning and discussion need to focus. For example, the value of respect in the case study in Box 2 prompted a reflection on whether the status of “employees” ever justifies preferential access to vaccination over “citizens”.

Because of potential discrepancies between values at personal and organizational levels, it is important for individuals to have a good understanding of what organizational values intend and what the expectation is for them to behave in alignment with such values.^3^ The framing of company values in our model helps to “bring those values to life” by connecting with the more personal intuitive level. Better application of company values has the potential to increase the autonomy and competence of employees involved, as it has been shown to do in healthcare settings.^26^ We believe that by strengthening the alignment between company and personal values our employees can more easily assume the role of “moral agents” driving company decisions, which is also supported by the feedback received on our workshop exercises. Virtue-based training that emphasizes internal values rather than externally imposed rules, and focuses on the virtuous characteristics of staff, is expected to lead to responsible and exemplary behavior.^32,33^

From our experience, the application of company values to drive decision making reduces the need to systematically generate written instructions, such as new or more complex “standard operating procedures” (or SOPs), and could also potentially reduce the number and volume of existing instructions. For example, the company’s code of practice for scientific external engagement has been significantly reduced in length and complexity thanks to the introduction of the TRIP & TIPP values-based model.

There is also some evidence that when moral case deliberations involve actors in the healthcare setting, they can have a measurable positive impact on interpersonal relationships and increase their engagement with the ethical dimension of their work.^26^ This supports our model’s dynamics, and in particular the involvement of impacted teams in evaluating options. This may also be preferable to the practice of routinely escalating ethical questions to a specific or senior board for resolution without engaging the ethical judgment of the team that is dealing with the situation on the ground.

The model facilitates continuous improvements through experienced learnings. Step 5 of the model, “How did it go?”, calls for retrospective evaluation of the result and documentation of the lessons learned. In practice, we have created a repository of all cases so that future decision making may benefit from comparison with previous cases. Experience so far has demonstrated the critical influence of contextual factors, which can result in quite different decisions in cases that may on the surface seem very similar (for an example of this, see the case in Box 2). Finally, in common with the healthcare setting, the biopharmaceutical industry shares a patient-centered mission. However, unlike the healthcare sector, the “patient” in the biopharmaceutical context is not known at the time decisions are made; hence, the decision needs to consider *anonymous patients, research participants, currently healthy populations (in the context of vaccines), and very often future patients during drug development stages*. It has been shown that when “someone” is distant and in the future, it is more difficult to make ethical decisions than when this someone is here and now and will be directly impacted by the decision.^34^ This can also be related to the idea of ethical awakening coming from the “Other” where “ethics cannot be separated from leadership and leaders’ responsibility to Others”.^35^ Therefore, we believe that by bringing the patient dimension into the “proximity” where practical decision-making takes place – as we do by having the specific value of “Patient Focus” – our model helps to evoke the “otherness” in employees’ minds, thus getting them closer to bringing the patient-related values to the forefront.

### Limitations

Practical experience of the model we report here is limited, and there is a need to expand our case study database at GSK Vaccines. Gathering and analyzing more structured feedback from users – including information on how much and in what ways the tool helped them in their decision-making – will also supply information about how the model is applied in real life and the quality of the outcomes.

Our assessment of the medium-term impact of the training is limited by the small size of the data set. Although response rates to our six-month survey seem low, at 33% for the face-to-face and 17% for the virtual, they are in fact of a similar order to average response rates reported in the literature for online surveys in evaluating educational courses.^36^ The higher response rate for the face-to-face workshops suggests these are more engaging than the virtual sessions. We are building on the experience from these preliminary surveys as a basis for designing a larger scale and more in-depth and structured survey to enable a robust assessment of our model and its use.

It could be argued that the values in our model do not provide clear moral guidance for action – a common criticism of principlism,^37^ which also refers to decision making by applying the four biomedical ethics principles developed by Beauchamp & Childress (non-maleficence, beneficence, autonomy, justice).^38^ As with principlism, the values in our model are specified, weighed and related to each other according to the specifics of the situation and organization. But in our model, moral guidance is further strengthened by the use of framing questions related to values and contextual factors, and the structured five-step approach.

Further, our model is not an automated tool based on algorithms; it relies on human judgment, which is prone to subjectivity resulting from differences in individual and personal understanding. In our approach, this risk is mitigated by the involvement of a neutral facilitator who has a relevant understanding of the business and bioethics but has no direct interest in the decision outcome. Furthermore, the multidisciplinary group assessment mitigates against subjectivity because of the many different perspectives represented.

There is a theoretical risk that the model could be (ab)used to justify a pre-chosen or preferred option. Related to this is the risk that certain values or preferences (e.g., consensus) may be given undue prominence in order to achieve a pre-chosen outcome. Such biases are avoided by giving priority to the value of patient focus. It is also important to gather and then document the consideration of quality data (facts, laws, regulations) to achieve the highest quality of generated options. Furthermore, there is the opportunity for continuous improvement and learning to increasingly avoid bias.

Finally, a further limitation is the likelihood of bias resulting from the decision-making team being company employees, with no independent actors. In fact, our model has been specifically developed for internal decision making because of the need to generate applicable solutions to the specific biopharmaceutical context, that is, R&D. Although it does not foresee systematic recourse to external (bioethics) expert advice or panels,^39^ it does not preclude asking for or including such knowledge as appropriate. In some cases, specific questions may require input from independent stakeholders, for example, patient and/or ethical advisory groups.

### Conclusions and way forward

This is in our opinion the first publication describing the implementation and early assessment of a values-based decision-making model in the biopharmaceutical industry. Our model brings an innovative approach through the integration of company values into option assessment, which guides and actively engages employees in the practical decision making. It has been applied within vaccines R&D scope but, as it provides a set of decision tools that can be applied by anyone in the line of accountable decision making, its application can be extended to other areas where business-related decisions impact patients.

We intend to further develop the model with more prospective case studies and refine its assessment, including impact on reduced length and number of written control standards (SOPs) in applicable areas. We plan to gather data regarding how often teams are using the model and how useful it is in their decision making; and to investigate which parts of the model the teams find to be clear and reliable and which need refinement and/or additional education. Equally, we would like to explore whether the methodology can be validated by comparing the examinations of similar cases by different teams to see if they arrive at similar decisions and/or lessons learned. By sharing our model, we encourage its application by other companies and also by stakeholders such as healthcare providers, patients, and investigators, which would be expected to contribute to the continuous refinement of the model and its application.

**Box 1.** Case study 1: Use of the model in human subject research.

KNOW the Situation: During a phase 2 clinical trial that is investigating the efficacy and safety of a new product, several participants become imprisoned. According to the study protocol, these participants should be considered as “lost to follow-up” because the prison authorities do not allow them to attend the study visits. There are international guidelines on research with prisoners but not specifically on how to manage research participants who become imprisoned during research. No internal sponsor policies or procedures describe what to do in this situation. Key stakeholders are identified to be the imprisoned study participants, study site staff, study sponsor and prison authorities. The underlying ethical question was presented as: “*If study participants become imprisoned during a study, is it unethical to keep them enrolled in the study, given that they have become a vulnerable population, even if there may be a personal benefit for them to continue to be part of the follow-up study?* EXPLORE: A number of options were generated by the study team. Maintain the study participants in the trial with their consent and complete the trial as planned with logistic adaptation, such as site staff to do study visits in prison.* Stop trial participation completely and consider the imprisoned participants as lost to follow up. Negotiate with the prison authorities to be permitted to monitor the participants’ safety and complete the vaccination schedule in prison. Negotiate with the prison authorities to be permitted to monitor the participants’ safety but do not complete the vaccination schedule in prison as it is a phase 2 trial where benefit of vaccination has still not been established. *Note that the first option was eliminated from further evaluation for logistical reasons – the prison authorities did not permit site staff visits, making it impossible for the site staff to travel to the prison to continue with full study procedures. MATCH: Examples of questions considered for TRIP & TIPP assessment are indicated in the table below.	
Transparency	How will we communicate the decision to study participants, staff and prison authorities?
Respect	How can we treat the people in prison on a parity with other participants in the study? Should they not be given the opportunity to remain in the study? Would the prison authorities allow the study staff to contact the people in prison?
Integrity	Can the continued participation of prisoners in the study be justified, in line with laws, policies, regulations and your own personal beliefs?
Patient Focus	How can the safety follow-up in the study be done successfully? Are imprisoned people still free to make a decision without pressure of coercion?
Timing	Are the sites able to make timely visits to the participants in prison to maintain them in the trial? Do they have sufficient time and resources to do so? When is the right time to amend the protocol?
Intent	Is the intention to keep the prisoners in the study clearly identified? Is it for research or safety follow up? Do we want to do this for the right reasons?
Proportionality	Would the chosen solution represent a burden on prisoner or study staff? Will it be acceptable for prison authorities? What other risks may it create?
Perception	Will the solution raise concerns over conducting clinical trials with vulnerable people?
GO FOR IT: Assessment with TRIP & TIPP guided toward the third and fourth options for the given situation and after team deliberation the fourth option was chosen as way forward. HOW DID IT GO?: Local ethics committees agreed with the proposal to continue the safety follow-up of the study participants in prison, which was done by telephone. Several participants “returned” to the study after they were released from prison within the one-year follow up period of the study.

**Box 2.** Case study 2: Use of the model for the assessment of vaccine access.

KNOW: It is a company policy at GSK Vaccines to offer vaccines to employees when they are licensed. However, due to a supply situation a new vaccine (“Vx”) for preventing shingles disease is not available in certain countries where it has been licensed. If Vx were to be given to employees in such countries, it would put them in a differential position with regards to the rest of the population. Hence, the underlying ethical question is: “Is it acceptable to offer Vx to any employees in countries where Vx is licensed but not launched, resulting in the situation that company employees have access to Vx, while other citizens (including their family members and friends) in these countries have no access?” EXPLORE: A binary choice of options was generated. It is acceptable. It is not acceptable. MATCH: Questions considered for TRIP & TIPP are indicated in the table below.	
Transparency	How will we communicate the chosen solution to employees and their families, and respond to questions coming from citizens in these countries?
Respect	Will we create inequality with other citizens, including families and friends of company employees (as they do not have access to the Vx)?
Integrity	How could you explain to your family members that they cannot get the Vx, even if eligible according to product recommendations? Is the chosen solution going to be in line with company policy for access to Vx by employees? What measures will need to be put in place to ensure compliance with local laws? Are the rights of employees respected?
Patient Focus	Which patients first? Are employees any different from other potential vaccinees? Will there be a risk of limited pharmacovigilance and adverse event reporting if employees go outside of the company for any concerns, e.g., treatment of side effects?
Timing	Do staff need to be vaccinated immediately (e.g., in the case of a current/imminent pandemic) so that they can keep patients protected? Is the vaccine to be made available in the country soon, hence the staff would only be getting early access to a product that the population will be getting in due course?
Intent	Is it the intention, in offering the vaccine to employees only, to create a privilege for company employees over the citizens in the country where the Vx is not available?
Proportionality	Would it be acceptable for employees working in countries where the vaccine is not available to travel to get the vaccine from an office in countries where it is? Would permission be needed from authorities to bring doses in foreign pack in the absence of a formal price and to translate prescribing information leaflet based on each country’s language? Is the effort of implementing such a program proportional to any benefit expected?
Perception	Will there be a perception that employees are being treated better than the rest of the population?
GO FOR IT: following the application of TRIP & TIPP to this particular situation, a consensus was reached to deviate from GSK Vaccines’ policy by not supplying the new vaccine to employees in the country in which it was not available to citizens living there. HOW DID IT GO?: Note the importance of context for the decision – the analysis for this Vx was compared to a previous decision in which a flu vaccine was given preferentially to employees (before being made available to the rest of the population) in the context of a declared pandemic by WHO. The earlier decision was justified by the need for the employees to be able to fulfil their role in order to ensure continuity of vaccine production and supply for the rest of the population. But in the current case of Vx the additional factor of urgency for employees to be vaccinated was not present, therefore the different decision was considered justified.

**Box 3.** Retrospective case: Children of minor parents in clinical research.

We used our model to retrospectively assess an R&D position that had been taken previously and published.^40^ The questions and answers generated during retrospective assessment are shown in the table below (the answers were derived from the position paper). The outcome of this exercise supported the decision that was made at the time. It also suggested that prospective use of the model could have facilitated the deliberations with the experts. KNOW: Children of minor parents are under-represented in clinical trials. This is due, in large part, to the ethical, legal, and regulatory complexities in the enrollment, consent, and appropriate access of children of minor parents to clinical research. Can these children be safely and ethically included in clinical research? EXPLORE: A binary choice of options was generated. Children of minor parents should be included in clinical research. Children of minor parents should not be included in clinical research. MATCH: Questions considered for TRIP & TIPP are indicated in the table below.	
Transparency	How will we inform the stakeholders of the intention to include children of minor parents in the clinical trial? Where will it be documented? *Answer*: The community must be engaged to establish the most sensitive way to implement the decision, respecting cultural norms. Then, it must be described in the study specific documentation (e.g., protocol), which needs to be approved by local Ethics Committees and Authorities.
Respect	How can we treat children of minor parents as equally as possible compared to other children, offering the benefits that other children receive from being enrolled in the trial yet ensuring they also benefit from the same level of protections, e.g., in terms of validity of informed consent? How can we best protect the rights of minor parents and their children? *Answer*: The emerging capacity of adolescents for autonomous decision making and respect for their role as a parent need to be balanced against the need for special protections due to their minor status. Like all parents, it is assumed that the minor parents and the legally acceptable representatives for the children of the minor parents act in the best interest of their children and as such are the best judges of whether their participation in the trial is warranted. Best evidence says that capacity for decision making of adolescents is similar to that in adults when sufficient time is taken to explain clinical trials, the specific study, and to obtain consent/assent in a situation free of emotions or distractions. Hence trial context is important: for example, obtaining informed consent in trials with very sick children may be more challenging than in vaccine trials with healthy children.
Integrity	What does the law say, if anything, on clinical research with children of minor parents? What are the regulatory requirements? What are the local practices in the absence of laws and regulations? What additional measures may need to be adopted to ensure valid informed consent? *Answer*: On a personal integrity level, it would not be justifiable to exclude children of minor parents from clinical trials *a priori*, if they can be enrolled legally, safely and ethically. Legal age for consent in research must be observed, or in its absence the legal age for consent to medical treatment. Local laws on consent related to minor parents or minors must be followed or in their absence local traditions, culture and common practices, and approved by the appropriate ethics committee(s). Additional measures must be considered to appropriately deal with the possible vulnerability of the minor parents and their children.
Patient Focus	Are the risks for children of minor parents different from other children? How can we engage minor parents maximizing benefits and minimizing harms for both themselves and their children? *Answer*: Children have the right to have decisions taken in their best interests, and to the highest attainable standard of health. Children of minor parents are more vulnerable than other children in the sense that they may experience greater rates of poverty, and as a result would be more likely to suffer diseases associated with poverty, so that exclusion from access to new treatments for diseases of poverty will affect them disproportionately.
Timing	When is the right time to decide on inclusion/exclusion of children of minor parents in the clinical trial? *Answer*: Before the start of the clinical trial in the country, all legal and cultural aspects around inclusion of these children must be investigated and the decision recorded in the trial protocol.
Intent	Is the intention to treat all children equally? Do we want to do this for the right reasons? Should this solution be applied to all clinical trials or be looked at on a study case basis? *Answer*: The intention is to treat all children equally, and to ensure that clinical trial populations reflect the intended vaccine recipient populations in order to obtain results robust enough to base future public health policy decisions on. This favors including children of minor parents in our clinical trials provided this can be done legally, safely and ethically.
Proportionality	Does the solution represent a disproportionate operational burden for limited benefit? What other risks may it entail? *Answer*: There is additional work associated with investigating the local legal framework and additional measures to ensure informed consent and assent is obtained in a locally appropriate way. Study or site-specific additional risks need to be addressed on a case by case basis. But this effort is not disproportionate, given the number of children involved and the importance of their having access to a new and potentially beneficial vaccine, and for the generation of reliable clinical trial evidence.
Perception	Could there be a perception of conducting clinical trials with vulnerable children if they are included? On the other hand, could there be a perception of discriminating against children of minor parents or taking the “easy” solution if they are excluded from clinical trials? *Answer*: Reasons for including children of minor parents in clinical trials must be clearly communicated and documented, as well as any additional measures taken to ensure their legal, safe and ethical inclusion.^40^
GO GOR IT: Guidance resulting from the retrospective use of TRIP & TIPP was in line with current recommendation, i.e., children of minor parents should be included in clinical trials, since exclusion would raise issues of appropriate access and equity. Minor parents should be involved in making decisions on research consent for their children. There are circumstances in which consent of an additional adult may be appropriate.^40^ HOW DID IT GO?: The result from the retrospective application of the model is in line with the guidance offered by Ott et al.,^40^ which has now been adopted as the recommended position on the topic.	

## Supplementary Material

Supplemental Material

## Notes

1 We emphasize here a distinction between “values-based” and “value-based”. The term “value-based decision making” is widely used within the healthcare and business environments, and although there is no universally accepted definition, it frequently refers to considerations of financial or commercial nature (example: NHS England Value-Based Decision Making, https://www.england.nhs.uk/south/wp-content/uploads/sites/6/2016/09/value-based-decisions.pdf). In our article, we use the term *values*-based to make it clear that we refer specifically to organizational *ethical* or *mission* values.

2 https://uk.gsk.com/en-gb/careers/working-at-gsk/our-culture-and-values/.
