# Supplementary material for: Ethical decision-making in biopharmaceutical research and development: applying values using the TRIP & TIPP model
Source: Hum Vaccin Immunother. 2020 Jan 15;16(8):1981–8. doi: 10.1080/21645515.2019.1700714 (PMC7482740; doi:10.1080/21645515.2019.1700714)
Supplement: Supplemental Material [file KHVI_A_1700714_SM2290.docx]

# Supplementary material

Terms used for literature searches to find research into decision-making models used in the biopharmaceutical industry.

Search engines used: Scopus, Embase, PubMed.

|  | AND | AND | AND | AND | AND |
| --- | --- | --- | --- | --- | --- |
| bioethic* / ethic* | “decision making” | tool / model / framework / strategy /  method | drug /  pharmaceutical / biomedic* /  biotech /  biopharma* /  bioindustry /  bioscience /  vaccine | industry / sponsor / corporate /  organi*ation /  company | research /  “R&D” |
